# Distinct Shades of Adipocytes Control the Metabolic Roles of Adipose Tissues: From Their Origins to Their Relevance for Medical Applications

**DOI:** 10.3390/biomedicines9010040

**Published:** 2021-01-05

**Authors:** Annie Ladoux, Pascal Peraldi, Bérengère Chignon-Sicard, Christian Dani

**Affiliations:** 1 Université Côte d’Azur, CNRS, INSERM, iBV, 06107 Nice, France; peraldi@unice.fr (P.P.); dani@unice.fr (C.D.); 2 Department of Plastic and Reconstructive Surgery, Université Côte d’Azur, Pasteur2 Hospital, 06107 Nice, France; chignon.b@chu-nice.fr

**Keywords:** adipocytes, adipose progenitors, white adipose tissue, brown/beige adipose tissue, breast adipose tissue, epicardial adipose tissue, thermogenesis, cancer, regenerative medicine

## Abstract

Adipose tissue resides in specific depots scattered in peripheral or deeper locations all over the body and it enwraps most of the organs. This tissue is always in a dynamic evolution as it must adapt to the metabolic demand and constraints. It exhibits also endocrine functions important to regulate energy homeostasis. This complex organ is composed of depots able to produce opposite functions to monitor energy: the so called white adipose tissue acts to store energy as triglycerides preventing ectopic fat deposition while the brown adipose depots dissipate it. It is composed of many cell types. Different types of adipocytes constitute the mature cells specialized to store or burn energy. Immature adipose progenitors (AP) presenting stem cells properties contribute not only to the maintenance but also to the expansion of this tissue as observed in overweight or obese individuals. They display a high regeneration potential offering a great interest for cell therapy. In this review, we will depict the attributes of the distinct types of adipocytes and their contribution to the function and metabolic features of adipose tissue. We will examine the specific role and properties of distinct depots according to their location. We will consider their cellular heterogeneity to present an updated picture of this sophisticated tissue. We will also introduce new trends pointing out a rational targeting of adipose tissue for medical applications.

## 1. Introduction 

Adipose tissue is the most expandable tissue of the organism as it plays a crucial role in controlling energy storage and release in the vertebrates. Besides its physiological function, it has a mechanical function as a cushion and insulates the body from cold and heat. This tissue extends when the energy intake is more important than the energy expenditure. An excessive fat storage leading to overweight and subsequent obesity participates to health disorders. Risk factors including abnormal plasma cholesterol and/or triglyceride levels, excess of body fat around the waist, high blood sugar, and hypertension promote the acquisition of the metabolic syndrome with an increased probability of heart disease, stroke, and type 2 diabetes [1]. In addition, a link between obesity and a high cancer incidence exists for many types of cancers [2]. As more than 1.9 billion individuals were considered overweight by the World Health Organization in 2016 (https://www.who.int/news-room/fact-sheets/detail/obesity-and-overweight), the obesity epidemics is a major Public Health concern [3]. On the other hand, a group of genetic or therapeutically induced disorders called lipodystrophies [4], whereby the body is unable to produce healthy and functional fat mass, preventing then lipid storage, recapitulates also features leading to metabolic syndrome development.

Adipose tissue is widely distributed all over the body where it enwraps most organs that instead have a precise location. Subcutaneous adipose tissue (SAT) is mainly located in the buttocks, the tights, and the abdomen. Inside the abdomen, adipose tissue is present in three prevalent locations i.e., attached to the stomach (omental), to the intestine (mesenteric) and, behind the kidneys (retroperirenal) [5]. Other organs host adipose tissue such as the bone marrow and the breasts. It also resides in the face, around the heart (pericardial), or the vessels (perivascular and periarterial). These many locations suggest that all depots are not equivalent. Adipose tissue is composed of many cell types organized as structural units called lobules. Adipocytes, i.e., cells presenting lipid droplets where lipids are stored as triglycerides, represent the major cell type. In addition, adipose progenitors (AP), pericytes, blood vessels, nerves, and various immune cells constitute the stroma vascular fraction (SVF), which is crucial for adipose tissue development and adaptation to metabolic constraints [6]. These processes mainly rely on the adipose immature stromal cells that retain characteristics of mesenchymal stem cells. They are endowed with an extensive regeneration potential that has drawn a special consideration for cellular therapy and regenerative medicine.

A better knowledge of the adipose tissue structure, cellular composition and function may deliver innovative approaches to improve adipose tissue diseases, restore correct metabolic function, and facilitate reconstructive strategies for medical or aesthetical purposes. Here, using examples, we will discuss and outline how distinct types of adipocytes contribute to the function and metabolic features of human adipose tissue. We will consider the specificity of the depots according to their location, and the heterogeneity in cellular composition to propose an updated picture unfolding the complexity of this tissue. We will also introduce new trends pointing out a rational targeting of adipose tissue for medical applications.

## 2. Distinct Adipose Tissue Functions Are Supported by Different Types of Adipocytes

### 2.1. White Adipose Tissue and Adipocytes

For long, white adipose tissue (WAT) has been known as a dynamic lipid storage tissue able to remodel the size of the cells to comply with the metabolic demand and to control energy homeostasis. WAT is present in many intra-abdominal depots and subcutaneously. Adipocytes are involved in this process to avoid ectopic fat storage that results in the metabolic syndrome [7]. Because fatty acids and their metabolites can be toxic, lipids are stored as triglycerides in a single fat vacuole that occupies the whole cytoplasm of the adipocyte and is surrounded by specific proteins, perilipin 1 being the most abundant [8]. They are released upon fasting. Storage of an excess of energy relies on two mechanisms: adipocyte hypertrophy (increase in size) and hyperplasia (increase in number). Then, the adipose tissue mass expands leading to overweight and obesity. Several processes are involved in this adaptation such as adipogenesis, lipolysis, and lipogenesis. In addition to this property, adipose tissue is endowed with endocrine function through secretion of adipocytokines, which control important physiological functions such as appetite, metabolism, immune response, or reproduction [9]. Leptin, which is involved in the hypothalamic regulation of food intake, was first identified in 1994 [10]. Adiponectin plays a crucial role in controlling glucose levels and fatty acid oxidation [11,12]. Other cytokines such as apelin, visfatin, and cytokines related to the immune system, i.e., interleukin-1β (IL-1β), interleukin-6 (IL-6), tumor necrosis factor-alpha (TNF-α), and monocyte chemotactic protein-1 (MCP-1) are also produced by adipocytes under certain circumstances [13,14]. During obesity, a state of chronic inflammation is associated with a modification of the level of production of these adipokines resulting in the development of a state of insulin resistance associated with this syndrome.

### 2.2. Brown Adipose Tissue and Adipocytes

Besides WAT, an adipose tissue with opposite metabolic function, i.e., the brown adipose tissue (BAT) exists. It does not store, but dissipates energy in a heat-producing process called thermogenesis, the primary source of energy being fatty acids. This process is involved in a proper maintain of the body temperature. In humans, BAT depots are found in fetuses, and for long, it was considered to be limited to newborns in perirenal, periadrenal, axillary, and cervical regions [15]. After birth, it atrophies and its presence rapidly declines but depots remain preferentially in the upper part of the body, i.e., in the supraclavicular region, in the upper part of the body (neck and face [16,17]), and around deep organs (such as the kidneys [18]). It was traditionally considered insignificant in adults, except for subjects exposed to cold climates for a long time or those affected by pheochromocytoma. BAT depots are metabolically active as they can respond to cold or to catecholamines stimulation. Note that sexual dimorphisms were reported for BAT depots in humans, but without any significant impact on its activation [19].

Brown adipocytes display a different morphology as they own multiple fat vacuoles and abundant mitochondria in their cytoplasm. Dissipation of energy occurs through the uncoupling protein 1 (UCP1), which uses the mitochondrial proton gradient to produce heat instead of ATP [20]. Indeed, the mitochondria-enriched adipocytes are active as they are able to respond to cold exposure and to βadrenergic stimulation through β3-adrenergic receptor activation. As WAT, BAT is a secretory organ. Its secretory profile is quite distinct from WAT, although BAT secretes classical adipokines such as leptin. Batokines, i.e., adipokines secreted by BAT, comprise fibroblast growth factor 21 (FGF21), IL6, bone morphogenic protein 8b (BMP 8b), and endothelin 1 [21].

### 2.3. Beige Adipose Tissue and Adipocytes

Recently, a new class of adipocytes, called beige (or brite) adipocytes was described. They display properties of brown adipocytes, but they are located within WAT depots. Note that in contrast to brown adipocytes, the expression of UCP1 is not constitutive in beige adipocytes, but inducible [22]. In addition to this unusual location for thermogenic adipocytes, they differ also from brown adipocytes in their developmental program as these adipocytes derive from white adipocytes. Upon stimulation, these cells are able to dissipate energy and to produce heat, like observed for brown adipocytes. WAT browning occurs almost exclusively in subcutaneous (SAT) depots in humans [23] which is another difference within the WAT depots. These adipocytes share common secreted batokines with BAT such as FGF21. Indeed, some adipokines are preferentially expressed in beige cells, such as Meteorin-like, which contributes to an increase in beige thermogenesis in mice [24].

WAT browning represents a promising therapeutic strategy to combat obesity, owing that this mechanism occurs in obese patients upon stimulation with an efficient and well tolerated drug that remains to be identified [25].

### 2.4. Breast Adipose Tissue and Pink Adipocytes

Breast is composed of epithelial ducts associated with adipose lobules and the adipose tissue is the major contributor to the volume of the breast, and it plays a crucial role in the morphogenesis of mammary glands. Within the breast, special female-specific adipocytes were described and called “pink adipocytes.” During pregnancy, the size of mammary adipocytes increases to store lipids [26,27]. Lipogenesis stops when lactation starts. Then, mammary adipocytes trans-differentiate into secretory epithelial cells to promote lipid transfer during milk production. This process participates actively to breast remodeling as breast adipocytes display an extraordinary plasticity. Breast adipose tissue is a secretory organ like the other depots. It produces prolactin and steroid hormones like estrogens thanks to an aromatase. In addition, it participates in epithelial cell growth, angiogenesis, intercellular communication, and milk production [28], and it secretes many growth factors and enzymes involved in beast reshaping during development [29]. Distinct endocrine features can also be exemplified as follow. For instance, WAT in the breast and also in the buttocks is sensitive to estrogens, in contrast to WAT in the upper back which is more sensitive to glucocorticoids [28,30]. This unique association between adipose tissue and the mammary epithelium outlines functional differences with other body fat depots.

**Figure 1:**
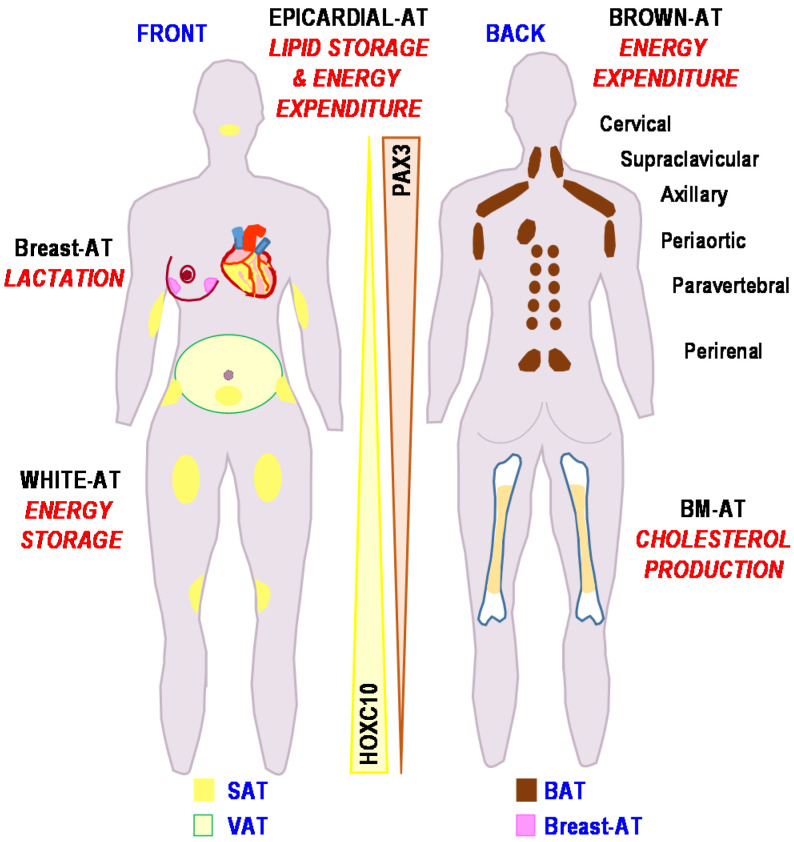
Locations of the adipose depots associated with their functions.

All together, these simple observations raise an important notion regarding adipose tissue biology. Depending on their location all over the body, the distinct fat depots harbor different adipocyte populations with proper functional characteristics that are recapitulated in Figure 1.

Schematic representation of the fat depots locations in the human body is shown in Figure 1. The main metabolic role of each adipose fat pad is mentioned. The inverse expression gradient for *PAX3* and *HOXC10* is indicated. (Adipose tissue (AT), subcutaneous adipose tissue (SAT), visceral adipose tissue (VAT), Brown adipose tissue (BAT)).

## 3. Distinctive Features of Some WAT Depots

Although the role in energy storage and release is well established for WAT, these many locations point out the existence of peculiar endocrine or metabolic features and one should consider that the intrinsic functions of the distinct depots are related to these specificities.

### 3.1. Visceral (VAT) and Subcutaneous WAT Depots

The physiological importance of WAT is specially underlined in two opposite situations, i.e., obesity characterized by an excess of adipose tissue and lipodystrophy (and/or lipoatrophy), which corresponds to generalized or partial adipose tissue deficiency that depends on the degree and the location of fat loss [31]. For instance, mutations in genes important for adipose differentiation such as peroxisome proliferator-activated receptor-*γ (PPARγ)*, or protein kinase B (*AKT2)* as well as genes essential for lipid droplets structure and lipolysis such as perilipin 1 (*PLIN1)* cause fat loss mainly in the subcutaneous adipose tissue from the extremities [4,31]. This last observation reinforces the idea of fat depots disparity.

In both situations, an excess of circulating fatty acids that contributes to ectopic fat accumulation and to insulin resistance is observed and it participates to the development of the metabolic syndrome. Together with muscles and the liver, WAT belongs to the insulin-responsive tissues through binding and activation of specific receptors. Insulin displays two main functions in white adipocytes. First, it inhibits lipolysis, a process which is exquisitely sensitive to insulin in adipocytes. Inhibition of triglyceride lipolysis decreases the postprandial plasma concentration in nonesterified fatty acids (NEFA). This is crucial to avoid inappropriate fat storage leading to lipid overload and ectopic fat deposition with its many adverse effects. Second, it increases glucose uptake and participates to regulation of glycemia. Insulin resistance is accompanied by aberrant high concentrations of plasmatic NEFA, together with a reduction in the glucose transporter 4 (GLUT4) translocation that attenuates glucose metabolism along with biosynthesis, esterification, and sequestration of fatty acids (i.e., lipogenesis) [32]. This situation is mainly encountered in poorly controlled type 2 diabetes with the risk of cardiovascular disease development.

A higher rate of lipolysis was observed in the visceral (omental) adipose tissue as compared to the subcutaneous (femoral/gluteal) adipose depots more than 20 years ago [33]. This may result from distinct sensitivities to beta-adrenergic stimuli or to insulin, which exerts a predominant antilipolytic effect in vivo. No clear role could be assigned to lipases and proteins associated to lipid droplets in this observation, except for an obese situation [34]. VAT and SAT display as well heterogeneity in free fatty acid uptake, VAT being more efficient than SAT in humans [35]. Differential adipokines production has been observed for VAT and SAT. The intra-abdominal WAT display respectively an adipocytokine secretion profile related to inflammation and type-2 diabetes, whereas the subcutaneous WAT secretes less pro-inflammatory cytokines and more leptin [36]. Note that molecules involved in innate immunity and acute phase response are preferentially produced in VAT [37].

### 3.2. Bone Marrow Adipose Tissue

Among the adipose tissues, human bone marrow adipose tissue (BM-AT) has been far less studied than WAT and BAT albeit it represents more than 10% of the adipose mass. Its location is restricted to the bones. In contrast to other fat depots, the microenvironment of this tissue mainly consists in hematopoietic and skeletal mature or progenitor cells. This implies an active role in the regulation of hematopoiesis and bone formation. Indeed, bone marrow adipocytes (BM-adipocytes) appear as negative regulators of hematopoiesis by promoting quiescence of progenitor cells [38]. This tissue increases with age and people with prevalent vertebral fractures display higher mean BM-AT [39]. As for the other fat depots; human BM-adipocytes depots secrete adipokines [40]. Leptin has a positive effect on osteogenesis as it increases bone mineral density [41], while adiponectin acts as an anti-osteogenic agent [42].

BM-adipocytes are unilocular cells that do not express brown or beige markers even in animals or humans exposed to cold. Indeed, they exhibit a peculiar lipid metabolism. In contrast to subcutaneous adipocytes, these native adipocytes are devoid of lipolytic activity as they cannot release free fatty acids upon adrenergic stimulation. In consequence, they display a higher content in monoacylglycerol as well as in cholesterol [43]. In addition, they displayed higher glucose uptake than WAT, BM-AT being a major site of basal glucose uptake in humans, although they resist to insulin-stimulated glucose uptake [44].

### 3.3. Epicardial Adipose Tissue

Adipose tissue depots located around the heart do not correspond to an ectopic fat deposition. They were poorly studied for an access restricted to small scraps obtained after open cardiac surgery. Epicardial adipose tissue (EAT) is a visceral thoracic fat depot in direct contact with the myocardium and coronary arteries [45]. Its vascularization is issued from coronary arteries. It is composed of small adipocytes, presenting brown adipose tissue features through expression of UCP1. This thermogenic profile is intended to protect the myocardium from the occurrence of fatal ventricular arrhythmias in case of cold exposure. This tissue displays higher lipogenic and lipolytic rates as compared with other fat depots. Hence, EAT is more efficient to store or release lipids on demand [46]. As other fat depots, EAT releases adipokines and inflammatory cytokines that may play a role in the development of heart diseases. As compared to SAT, EAT is characterized by a marked expression of transcription factors associated with cardiac development and function. At this point, it is unclear whether this signature corresponds to a memory of the tissue location of if it plays a role in the cardiac function. EAT displays also an overrepresentation of immune-related genes expression. However, there is an inverse correlation between a high UCP1 expression and expression of immune-related genes, especially those correlated with T cells responses and the adaptive immunity. The number of genes with a modified expression in obese versus lean patients is scant [46]. It secretes more matrix metalloproteases, enzymes that remodel the extracellular matrix, as well. The heart is surrounded by another fat depot, i.e., the pericardial adipose tissue (PAT), which is located between the two pericardial layers with a vascularization that originates from the internal mammary artery [47]. In humans, an expansion of these tissues promotes the onset, the progression, and the severity of coronary arterial disease and atrial fibrillations. Thus, beneficial effects of their reduction on the management of coronary arterial disease or cardiac rhythm disorders need to be further investigated with randomized controlled studies.

### 3.4. The HOX Code: From Anatomical Organization to Function of Adipose Depots

Fat depots exhibit a signature linked to their anatomical location. They express distinct homeobox genes (HOX) depending on their position. These genes encode transcription factors that are crucial to direct the formation of limbs and organs along the anterior-posterior axis of the body during development. HOX genes are expressed as gradients. Their expression persists in adult tissues, including in adipose fat pads, and their expression profile delineates precisely their anatomical location. For instance, *PAX3,* which belongs to the PRD homeobox class named after the *Drosophila* gene *paired* [48] is a marker of neuroectoderm origin highly expressed in the upper part of the body as compared to the lower. An opposite situation is encountered for *HOXC10* as the main expression is observed in the lower part of the body [49]. Moreover, HOXA5 and NR2F1 were upregulated in abdominal SAT versus gluteal SAT [50]. The importance of this code for specific fat depot properties will be further discussed but remains to be formally established.

All together, these examples indicate that adipose tissues from distinct depots are not equal as they have their own specificity. The major differences concern lipid metabolism, the thermogenic ability, and the interactions with other systems. For instance, BM-AT is specialized in cholesterol production and is less prone to lipolysis than SAT. EAT is characterized by a general activation of immune-related pathways. However, high UCP1 expression in this tissue was associated with downregulation of genes involved in the production of reactive oxygen species and immune responses.

## 4. Distinct Adipose Progenitors Raise Fat Pads with Different Functions

**Figure 2:**
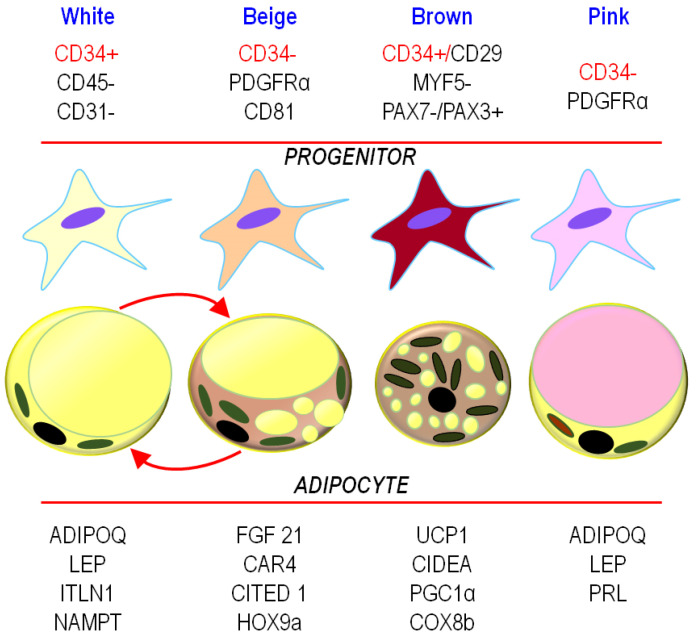
Adipose progenitors (APs), adipocytes, and their markers.

The diversity of adipocytes brings up the question of their origin. For long, adipose progenitors (AP) were not distinguished from fibroblasts. The only recognized marker for these cells was *Pref-1* (also known as DLK-1) [51]. *Pref 1* is expressed in both white and brown AP, and its expression decreases with the onset of differentiation. However, as adipocytes display distinct functions depending on the fat depot, one can wonder whether heterogeneous progenitor populations that contribute to the complexity of adipose tissue exist. These populations are presented in Figure 2.

In humans, adipose tissue development begins during gestation and both subcutaneous and visceral depots are present at birth. Nevertheless, adipose depots were shown to display distinct embryonic origins. While most of the adipocytes derive from mesoderm, lineage tracing experiments in rodents showed that adipocytes from the face and the neck derive from neuroectoderm [52].

The presence of adipose progenitors persists in adult adipose depots, providing them with the capacity to generate new adipocytes upon metabolic demand. The first human mesenchymal stem cells efficient to generate adipocytes in vitro were characterized as CD44+, CD49b+, CD105+, CD90+, CD13+, CD34neg, CD15neg, CD117 neg, Flk-1neg, CD133 neg for hMADS cells [53], or CD29/CD63/CD81/CD122/CD164 positive and CD34/CD36/CD45/CD117/HLADR negative for MIAMI (marrow-isolated adult multilineage inducible) cells [54]. Further molecular characterization of human cells isolated from the stroma vascular fraction of adipose tissue identified CD45−/CD34+/CD31− cells as mesenchymal progenitors [55]. These progenitor cells reside in two niches of the subcutaneous adipose tissue, i.e., the septa and the stroma. In addition, their characterization revealed that they were endowed with distinct adipose differentiation potentials, i.e., the highly adipogenic progenitors identified as CD36+/CD34+/MSCA1+ (i.e., *PPBT*+) were preferentially located in the stroma. In contrast, the MSCA1− progenitors displayed a fibrous phenotype with MSCA1−/CD271− cells in the septa and MSCA1−/CD271^high^ found in both compartments [56].

Recently, the heterogeneous pools of progenitor cells involved in adipose tissue development were analyzed by single-cell RNA seq [57,58]. Several populations with distinct adipogenic potential exist in the stromal vascular fraction of adipose tissue. In mice, the Lin-/CD29+/CD34+/SCA1+ cell population contains three subpopulations: the first one is enriched in cells expressing stem cells markers, the second one is enriched in cells expressing genes related to adipogenesis, and the third one, representing less than 10% of the cells, displays low adipogenic potential and is even able to inhibit adipose differentiation [58]. This last population was associated with blood vessels and characterized by CD142− and ABCG1-positive cells. More recently similar investigations were carried out on cells expressing common progenitor or mesenchymal cell surface markers including platelet-derived growth factor receptor-α (PDGFR-α), CD29, CD34, SCA1, LY6A, and CD24 [57]. Several groups of progenitors were identified including a group of cells expressing CD142 and another one expressing ICAM1 (intercellular adhesion molecule 1) that was described as a committed preadipocytes group. In addition, a group of interstitial progenitors expressing DPP4 was shown to generate CD142+ progenitor cells, ICAM1+ preadipocytes, as well as adipocytes. These data clearly identify distinct populations of functional adipose progenitors that obey a developmental hierarchy governing their fate. These populations were also found in human subcutaneous adipose tissue.

Schematic representation of the distinct types of adipocytes and their progenitors with their molecular signatures is shown in Figure 2. The list of genes expressed is not exhaustive and limited to those associated with characteristic features. The names of the genes are specified in the list of abbreviations. CD34 is indicated in red as its expression may vary depending on the microenvironment [59]. The other genes, whose expression is not dependent on the microenvironment, are written in black. The red arrows indicate a possible conversion of white into beige adipocytes.

### 4.1. Brown Progenitors

Distinct populations of adipose progenitors were shown to coexist within the same fat depot, which complicates the identification of specific progenitors for one subtype of adipocytes. In this regard, note that CD29 highly positive cells, which are preferentially encountered in the SVF isolated from human BAT as compared to human WAT, are prone to differentiate into adipocytes with great thermogenic potentiality [60]. In rodents, brown adipose progenitor cells were shown to derive from *myf5*-expressing muscle progenitors. Specialization into brown adipocytes was controlled by the transcriptional regulator PRDM16 [61], while its presence was not sufficient to maintain UCP1 in presence of browning inhibitors in human cells [62].

In humans, CD34+/CD146−/CD45−/CD56− cells isolated from fetal muscle, which did not yet express significant levels of *MYF5* mRNA, displayed great ability to differentiate into brown adipocytes in vitro, while CD34 + cells isolated in adults were less efficient [63]. Another study shows that CD34+/CD31− cells isolated from small vessels display the ability to differentiate into brown adipocytes [64]. Although it is tempting to consider that CD34 allows some discriminations between the different progenitor types, one should keep in mind that its expression may vary depending on the microenvironment [59] prohibiting its use as a reliable marker.

### 4.2. Beige Progenitors

Beige adipocytes derive from CD31−/CD45−/CD29+/CD34− cells isolated from the stromal vascular fraction prepared from human omental, subcutaneous, or gluteofemoral adipose tissue, while the CD34+ cells expressed less beige adipocytes markers (i.e., PGC1α, CITED1, TCF21, and UCP1) [65]. More recently, another origin for beige adipocytes was identified, i.e., the Lin-/Sca1/CD81+ progenitor cells in mice and Lin−/PDGFRα+/CD81+ cells in subcutaneous adipose tissue in humans [66].

### 4.3. HOX Genes in White and Brown Adipose Progenitors

Several *HOX* genes are detected in human fat depots. Lineage tracing in mice showed that brown adipocytes originate from Pax3/Myf5 myogenic progenitors [67]. PAX3 being a marker of stem cells with a neural crest origin, this observation is in line with lineage tracing results showing the neuroectodermal lineage of adipocytes present within the head and the neck of mice [52]. Indeed, in humans, *PAX3* is preferentially expressed in brown as compared to white fat depots and it is substantially enriched in the brown adipose progenitor population derived from human iPS cells [68]. In contrast, *HOXC10*, which is expressed in the lower part of the body where WAT depots are prominent and browning is scant, was reported as a suppressor of WAT browning in mice [69]. In addition, *HOXC8* and *HOXC9* genes are specific of undifferentiated white APs, hence these *Hox* genes are preferentially expressed in white adipose tissue [68]. *HOXC9* gene was not detected in brown APs, which is consistent with a “classical” brown phenotype [70]. In contrast, *HOXA5* was detected both in intra-abdominal fat depots in mice and in BAT adipose depots [71]. All together these examples indicate that the HOX code may not just stand for an “anatomical signature” but may also participate to the intrinsic properties of distinct adipose depots.

### 4.4. Bone Marrow Adipose Progenitors

Bone marrow contains multipotent mesenchymal stem cells that are able to differentiate into adipocytes, but also in osteoblasts depending on the modulators present in the environment. Comparison of cell surface markers between mononuclear cells isolated from human bone marrow and adipose tissue SVF showed that expression of CD34, CD105, CD73, CD90, CD166, and SUSD2 were significantly higher in adipose SVF [72]. Of note, the authors performed this study on crude cell populations that were not depleted of CD45- and CD31-positive cells. These discrepancies point out possible distinct functions between the adipocytes issued from these two locations. These cells also secrete factors involved in the hematopoiesis such as G-CSF, GM-CSF, and cytokines, i.e., IL-6, in vitro [73].

### 4.5. Breast Adipose Progenitors

In human breast, mammary stem cells that generate milk-secreting alveolar cells have been extensively characterized [74,75]. These progenitors were not shown to produce adipocytes and information on the putative breast adipose progenitors is scant. Bone marrow and breast adipose progenitors display a CD34-negative phenotype. Comparison of these fractions showed that the percentage of positive cells for CD10 and CD166 was significantly different, i.e., it was lower for the former and higher for the second in the bone marrow population. In addition, expression of CD24 was higher in bone marrow cells, in contrast to CD26 (DPP4), the neurofilament light polypeptide and the endoplasmin that were preferentially expressed in breast mesenchymal stem cells [76]. Indeed, both cell types neither expressed the endothelial marker CD31 nor the hematopoietic marker CD45. In lactating mice, CD31−/CD45−/PDGFRα+ cells isolated from mammary gland efficiently differentiate into adipocytes in vitro. These cells were shown to derive from adipocytes that lost their lipid content and regained preadipocytes features, through differentiation–dedifferentiation processes. Upon cessation of lactation, these cells proliferate and rise new mature adipocytes [27]. Human breast tissue displayed a high expression of *PAX3* and a low expression of *HOXC10*, although expression has not been measured directly in adipose progenitors [49]. A better knowledge of the mammary adipose progenitors deserves great interest because of their so special behavior/remodeling during mammary gland involution associated with gestation and lactation. Other important points concern the success and the safety of aesthetic and reconstructive surgery performed after mastectomy.

The identification of specific adipose progenitors for each adipocyte subset does not exclude that under appropriate conditions, white adipocytes acquired beige adipocytes features (beiging) and vice versa (whitening). This mechanism is reviewed in [77,78].

Heterogeneity among progenitors and adipocytes of the different fat depots is also observed in terms of response to pharmaceutical agents. Highly active antiretroviral therapy used to treat AIDS patients induces unwanted adverse effects, lipodystrophy being one of the most frequently noticed. HIV-protease inhibitors (HIV-PI) were shown to impair adipocyte differentiation [79,80]. However, adipogenic gene expression was preferentially decreased in SAT but not altered in VAT of AIDS patients treated with antiretroviral therapy, highlighting differences between these two depots in sensitivities to the treatment [81]. In addition, we recently showed that adipose depots from the upper and lower part of the human body, i.e., the chin and knee, were not equally altered. Lopinavir, one of the most prescribed HIV-PI, decreases self-renewal of chin adipose progenitors through disruption of the Activin A autocrine loop that is essential to maintain this process [82]. In knee adipose progenitors, lopinavir decreased preferentially adipose differentiation while self-renewal was less affected. These observations may contribute to identify pathways targeted by AIDS therapy and to explain, for instance, why it induces a loss of subcutaneous fat in the face.

## 5. Challenges for an Optimized Utilization of Adipose Tissue

### 5.1. Igniting Brown Fat Thermogenesis to Fight Obesity

Increasing pharmacologically the energy expenditure has been considered as a suitable method to fight obesity. Two alternatives can be used to reach this goal: either an activation of the pre-existing BAT or a conversion of white adipocytes into brown beige adipocytes. Some molecules able to activate BAT were identified. In a first attempt, stimulation of beta-adrenergic pathways was examined. Chronic treatment with the synthetic β3-adrenergic receptor agonist mirabegron, which is used to activate BAT in fluoro deoxyglucose positron emission tomography, enhances the resting metabolic rate in men and increases the BAT volume in women [83]. However, adverse unwanted effects preclude a chronic treatment at efficient doses to activate BAT.

Besides this drug, natural products were reported to potentially turn thermogenesis on. Many studies were conducted in rodents. However, their pertinence is questionable for several reasons, i.e., most often they are conducted in inbreed strains, brown adipose tissue is relatively abundant in rodent while it is scarce in human, and the metabolisms in human and rodents are quite different as well as the gut microbiomes. While several in vitro studies are reported, in vivo analysis of the phytochemicals-induced activation of BAT in humans is insufficiently documented.

Resveratrol is a natural antiaging polyphenol able to activate Sirtuin 1 and displaying an activation of browning [84]. We showed that this molecule induced UCP1 expression in adipocytes in vitro, through activation of p38 phosphorylation at concentrations lower than those required to induce *SIRT1* expression [62]. However, the efficiency of the body to modify this molecule into derivatives that are less efficient represents a major drawback for its use in humans [85]. Alkaloids such as capsaicin and berberine were also shown to induce browning of WAT. Loss of function experiments carried out in mice show the implication of the vanilloid receptor as a thermogenic mediator [86]. However, capsaicin ingestion seems to be less efficient than cold exposure (14.5 °C) to induce BAT activation in humans, while it increases energy expenditure as compared to controls [87].

More in vivo studies need to be carried out to ascertain of beneficial effects and safety of these natural products.

### 5.2. Regenerative Medicine

Mesenchymal stem cells and adipose-derived stem cells hold remarkable properties as for regeneration and therapeutic potential. Due to its accessibility and abundance, adipose tissue is widely used to for this purpose. Several techniques are proposed.

- Adipose-derived stem cells can be prepared from lipoaspirate and isolated after enzymatic or mechanical dissociation. They display tremendous properties, in particular, high self-renewal and differentiation potentials, angiogenic potency, ability to reduce immune and inflammatory responses… [88,89,90]. They were proposed to treat several disorders, including knee osteoarthritis, heart failure, or chronic myocardial ischemia that are currently subject to clinical trials [91].
- Autologous fat graft consists in harvesting adipose tissue where a surplus of subcutaneous fat is found to re-implant it where adipose tissue is missing. It represents a gold standard method for soft tissue repair and reconstruction, for instance, after mastectomy, to repair facial deformities after injury, illness, or congenital malformations. The major advantage is that tissue is re-implanted in the same individual, preventing then any immune rejection response. However, one can wonder if all tissue grafts are equivalent for long-term engraftment in a breast localization. Considering that fat depots are heterogeneous along the body, this method will lead to the relocation of mature adipocytes and adipose stem cells from a donor site to a recipient site holding a different microenvironment. For instance, we observe high expression levels of *PAX3* and low levels of *HOXC10* in breast adipose tissue, while the opposite expression is found in many donor sites such as knee or abdomen used for reconstruction surgery [49]. This raises the risk of misappropriation for the adipose tissue metabolic function.
- Another possibility would be the grafting of autologous fat pads from the same anatomical location, but grown ex vivo. As the above method, this technique would present the advantage of being allogenic preventing then any immune rejection. Note that the amplification process requires tissue culture, therefore, the methods and the culture media used need to be properly controlled to provide safe and reliable tissue implants.

### 5.3. Cancer-Induced Modifications of Adipose Tissues and Issues for Reconstructive Surgery

Although the importance of the tumor microenvironment for disease progression was shown to be crucial, the role of adipose tissue, which represents the most abundant tissue in the environment surrounding organs, has been neglected for long. The adipocyte-free fatty acids present in the adipose tissue represent an important source of lipids for cancer cells. However, adipocytes do not just stand as a reservoir of energy for cancer cells, i.e., they secrete adipokines that promote growth of cancer cells or even epithelial-mesenchymal transition [92]. They are more abundantly produced when adipose tissue expands. Therefore, the World Health Organization has classified obesity as a risk factor for several cancers, i.e., liver, kidney, multiple myeloma, meningioma, colorectal, ovarian, or breast cancers, among them. During cancer progression, adipocytes turn to be in direct contact with cancer cells. These adipocytes, which are close to the tumor tissue display smaller lipid droplets as compared to those remaining distant from the tumor [93,94]. They were called cancer-associated adipocytes and they exhibit delipidation due to an enhanced lipolysis. This process may be responsible at least, in part, for an accumulation of fibrous tissue associated with tumors.

Several molecules secreted by the tumor cells may be involved in this process such as catecholamines [95] or proinflammatory cytokines [96]. Note that in breast tumors, lipolysis can be accompanied by browning of adipocytes adjacent to the tumor tissue through adrenomedullin (ADM) secretion [94]. The implication of this metabolic modification for tumor progression remains to be understood. So far, few molecular signals involved in these bidirectional interactions between adipocytes and cancer cells were identified and more work is required to complete the list of factors implicated. Another important point is to figure out whether tumors modify only the neighboring adipocytes or if they secrete molecules able to act distantly. For instance, ADM expression is enhanced in the serum of patients with high-grade breast tumors and is correlated with lymph node metastasis [97]. However, ADM induces browning of adipocytes adjacent to breast tumor cells [94] or of fat pads adjacent to pheochromocytoma [98,99], pointing out a rather local effect. Distal ADM effects distinct from adipocyte browning cannot be excluded, but remain to be described. In contrast, systemic distal dysfunctions of adipose tissue have been reported for patients with multiple myeloma. Adipose stem cells (ASC) isolated from visceral subcutaneous tissue of multiple myeloma patients display an increased senescence and a defective osteogenic differentiation as compared to ASCs from healthy donors [100]. In multiple myeloma patients, both bone marrow mesenchymal stem cells and distal ASCs display similar alterations. This hinders the use of the distal ASCs for autologous transplantation to repair bones. These examples illustrate the complexity of tumor-induced adipose tissue modifications. They also address the issue of safe and efficient use of adipose tissue for fat grafting in plastic and reconstructive surgery.

So far, distinct studies conducted to evaluate the safety of autologous fat grafting for breast reconstruction after mastectomy did not point out either significantly enhanced local or systemic cancer recurrence or second cancer occurrence [101], supporting the reliability of this method. However, as adipose progenitors isolated from distinct fat depots display intrinsic differences, one should consider that the origin of fat grafts matters. The distinct embryonic origins and the molecular signatures associated with fat depots and adipose progenitors point out that a mismatch between the donor and the recipient sites may lead to opposite metabolic phenotypes. For instance, most of the donor sites used for breast lipofilling exhibit low levels of *PAX3* and high levels of *HOXC10*, while an opposite situation is encountered in breast [49]. Knowing that *HOXC10* is involved in tumor progression [102] and that adipocytes were reported to sustain proliferation and dissemination of cancer cells, the impact of adipocytes expressing high levels of *HOXC10* in promoting any residual breast cancer disease after reconstructive surgery requires crucial evaluation. In addition, whatever donor site is retained for breast lipofilling, the new adipocytes will not exhibit the plasticity of healthy breast adipocytes. It is difficult to propose a classification of the best matches between different donor fat sites and the breast environment. Choosing depots with similar molecular signatures would help to retain the metabolic phenotype of the depot, hoping that the influence of the new microenvironment will not deeply change it. Further investigations are necessary to clarify this point. Production of autologous fat spheroids suitable for transplantation might be an alternative to develop in the near future.

## 6. Conclusions and Future Inquests

Adipose tissue is the most scattered tissue in the body, but also the most diverse. All adipocytes contain lipid droplets, display some common markers such as PLIN1, leptin, adiponectin… but they emerge from different embryonic origin and are able either to store or to dissipate energy, two opposite functions. The lack (lipodystrophies) or an excess (obesity) of adipose tissue raise similar metabolic disorders as lipid storage is altered in both situations. Although the cardiometabolic risks linked to adipose tissue dysfunction are well studied, further investigations are required to better understand the role played by the different types of fat pads to support other diseases, such as cancer. In addition, this tissue has tremendous capacities in term of producing cells endowed with a high regenerative potential. More studies are necessary to evaluate the regenerative potential of the distinct fat pads to deliver safe outcomes for metabolic treatments and more efficient practices for regenerative medicine.

## Abbreviations

AP	adipose progenitors
ADIPOQ	adiponectin
AIDS	acquired immune deficiency syndrome
ASC	adipose stem cells
AT	adipose tissue
AKT	protein kinase B
BAT	brown adipose tissue
BM-AT	bone marrow adipose tissue
BMP 8b	bone morphogenic protein 8b
CAR4	carbonic anhydrase 4
CD29	integrin beta-1
CD31	PECAM1 or platelet endothelial cell adhesion molecule 1
CD34	hematopoietic progenitor cell antigen CD34
CD45	PTPRC or protein tyrosine phosphatase, receptor type, C
CD56	neural cell adhesion molecule 1
CD81	cluster of differentiation 81 or target of the antiproliferative antibody 1
CIDEA	cell death inducing DFFA like effector A
CITED1	Cbp/P300 interacting transactivator with Glu/Asp rich carboxy-terminal domain 1
COX8b	cytochrome c oxidase subunit 8
FGF21	fibroblast growth factor 21
GLUT4	glucose transporter 4
HOX9a	encodes for homeobox-leucine zipper protein HOX9
HOXC10	homeobox C10
HIV	human immunodeficiency virus
HIV-PI	HIV-protease inhibitors
ICAM1	intercellular adhesion molecule 1
IL-1β	interleukin-1β
IL-6	interleukin-6
iPS cells	induced-pluripotent stem cells
ITLN1	omentin
LEP	leptin
MCP-1	monocyte chemotactic protein-1
MIAMI cells	marrow-isolated adult multilineage inducible cells
MSCA1	mesenchymal stromal cell antigen-1 or alkaline phosphatase encoded by PPBT gene
MYF5	myogenic factor 5
NAMPT	nicotinamide phosphoribosyltransférase or visfatin
NEFA	nonesterified fatty acids
PAX3	paired box gene 3
PAX7	paired box gene 7
PDGFR α	platelet-derived growth factor receptor α
PGC1 α	peroxisome proliferator-activated receptor gamma coactivator 1-alpha
PPARγ	peroxisome proliferator-activated receptor-γ
PRL	prolactin
SAT	subcutaneous adipose tissue
SVF	stroma vascular fraction
TNF-α	tumor necrosis factor-alpha
UCP1	uncoupled protein 1
VAT	visceral adipose tissue
WAT	white adipose tissue
